# Non-invasive detection of anemia using lip mucosa images transfer learning convolutional neural networks

**DOI:** 10.3389/fdata.2023.1291329

**Published:** 2023-11-03

**Authors:** Shekhar Mahmud, Mohammed Mansour, Turker Berk Donmez, Mustafa Kutlu, Chris Freeman

**Affiliations:** ^1^Department of Systems Engineering, Military Technological College, Muscat, Oman; ^2^Mechatronics Engineering Department, Sakarya University of Applied Sciences, Serdivan, Sakarya, Türkiye; ^3^Biomedical Engineering Department, Sakarya University of Applied Sciences, Serdivan, Sakarya, Türkiye; ^4^Electronics and Computer Science, University of Southampton, Southampton, United Kingdom

**Keywords:** anemia, image processing, deep learning, classification, convolutional neural network (CNN)

## Abstract

Anemia is defined as a drop in the number of erythrocytes or hemoglobin concentration below normal levels in healthy people. The increase in paleness of the skin might vary based on the color of the skin, although there is currently no quantifiable measurement. The pallor of the skin is best visible in locations where the cuticle is thin, such as the interior of the mouth, lips, or conjunctiva. This work focuses on anemia-related pallors and their relationship to blood count values and artificial intelligence. In this study, a deep learning approach using transfer learning and Convolutional Neural Networks (CNN) was implemented in which VGG16, Xception, MobileNet, and ResNet50 architectures, were pre-trained to predict anemia using lip mucous images. A total of 138 volunteers (100 women and 38 men) participated in the work to develop the dataset that contains two image classes: healthy and anemic. Image processing was first performed on a single frame with only the mouth area visible, data argumentation was preformed, and then CNN models were applied to classify the dataset lip images. Statistical metrics were employed to discriminate the performance of the models in terms of Accuracy, Precision, Recal, and F1 Score. Among the CNN algorithms used, Xception was found to categorize the lip images with 99.28% accuracy, providing the best results. The other CNN architectures had accuracies of 96.38% for MobileNet, 95.65% for ResNet %, and 92.39% for VGG16. Our findings show that anemia may be diagnosed using deep learning approaches from a single lip image. This data set will be enhanced in the future to allow for real-time classification.

## 1. Introduction

**Figure 1:**
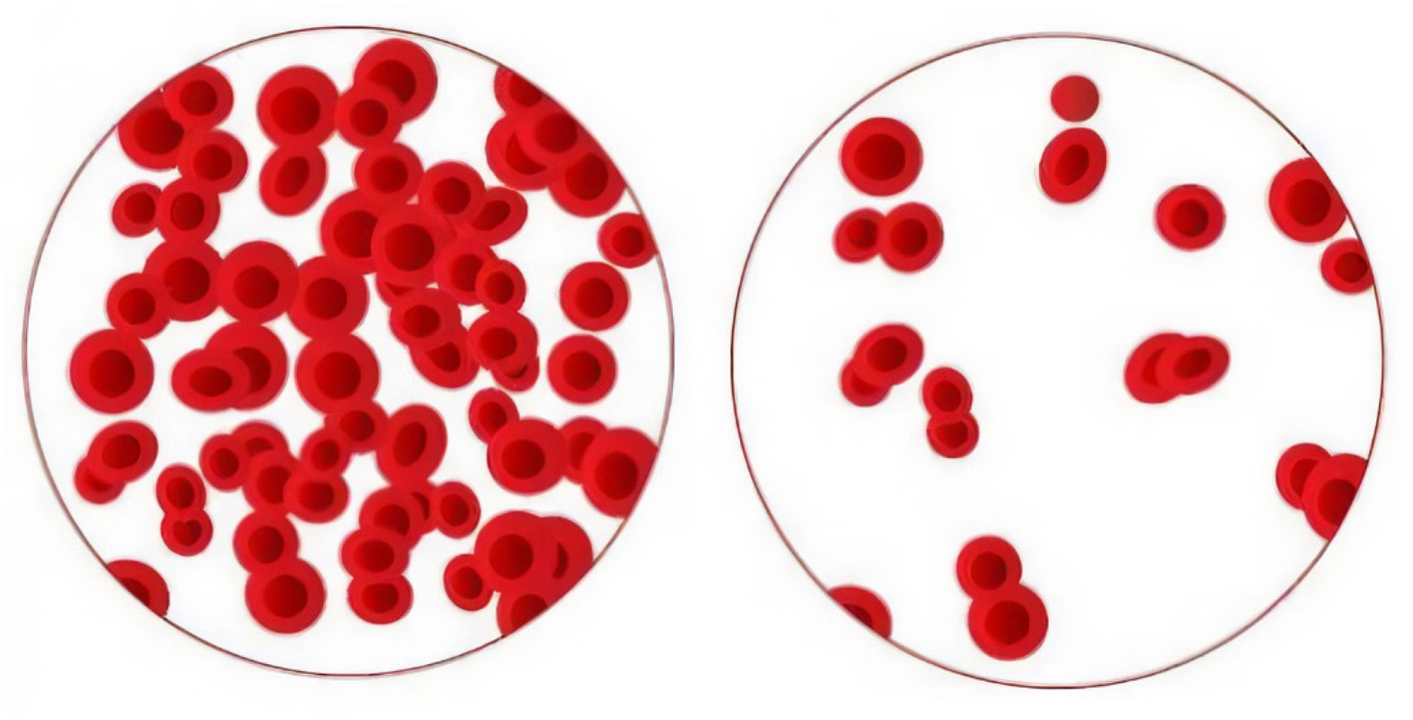
Healthy and anemia blood cells.

Anemia is a condition in which the number of hemoglobin-containing red blood cells in the blood is reduced. The World Health Organization (WHO) defines anemia as a hemoglobin level in the blood that is less than 13 g/dl in men, 12 g/dl in women, and 11 g/dl in pregnant women (Conrad, 2011). Anemia is most commonly caused by a decrease in red blood cell synthesis or an increase in red blood cell breakdown and loss (Brown, 1991; Aapro et al., 2002; Martinsson et al., 2014). Anemia can also be caused by the creation of defective red blood cells in some hereditary blood illnesses. As seen in Figure 1, this results in a drop in the average red blood cell count in the blood. Taking intravenous blood from a venous vein and examining it with a hemogram is the gold standard for identifying anemia (Prefumo et al., 2019; An et al., 2021; Milovanovic et al., 2022). Invasive operations are uncomfortable and difficult to coordinate, especially in pregnant and pediatric patients (Bashiri et al., 2003). The subject must visit a clinic to undergo the necessary procedure. Following the COVID-19 pandemic, executing these operations in medicine, as well as traditional follow-up measures, is no longer possible. Non-invasive anemia monitoring may bring benefits in terms of patient comfort.

Anemia presents with a variety of clinical symptoms, but one of the most prevalent indicators is skin pallor. Therefore, areas with thin skin, such as the conjunctiva, lips, tongue, and oral mucosa, are particularly suitable for diagnosis. For instance, Wang et al. (2016) used a smartphone app to non-invasively assess the hemoglobin concentration of blood, which makes use of the camera and various lighting sources. Tamir et al. (2017) determined anemia from the pallor of the front conjunctiva of the eye, Mannino et al. (2018) estimated hemoglobin levels from the color and metadata of smartphone photos shot on the nail bed, and Rojas et al. (2019) developed Selfie Anemia, a non-invasive smartphone app that estimates hemoglobin under controlled illumination. Non-invasive methods for anemia detection have been explored, with a focus on the conjunctiva in general (Dimauro et al., 2020; Rahman et al., 2020; Suner et al., 2021). Patients, on the other hand, will find it incredibly difficult to determine anemia from a conjunctival image using a simple phone camera for IoT connectivity. While there are benefits to diagnosing anemia non-invasively by taking pictures of the nail bed, hand, and conjunctiva, there are also certain restrictions as well. These restrictions include difficulties with accuracy brought on by differences in skin color, the effects of variables, the existence of medical problems, and patient variety. Problems also exist around data quality, privacy problems, associated expenses, approval procedures, and the requirement for clinical validation. It is crucial to keep in mind that any diagnostic method, including this one, should be used in conjunction with other tests and clinical judgment to achieve accurate findings. Effectively addressing these constraints necessitates study, exhaustive validation methods, and cautious implementation. Images of the lip mucosa can be used to diagnose problems in a special way. Since this procedure is non-invasive, patients won't experience any discomfort as a result of it. It is patient-friendly since it is simple to access and suited for low-cost screening programs. We can eliminate pain and potential infection risks connected with blood testing by evaluating the lip mucosa. Given that it encourages acceptance and engagement, this strategy is especially appealing to those who are afraid of needles or who work in healthcare environments. Its flexibility for continuous monitoring and use in certain populations, such as pediatric patients, emphasizes its promise as a practical and successful diagnostic strategy even more. The effectiveness of lip mucosa analysis in identifying diseases like anemia, however, has to be thoroughly demonstrated via scientific study and clinical trials.

**Figure 2:**
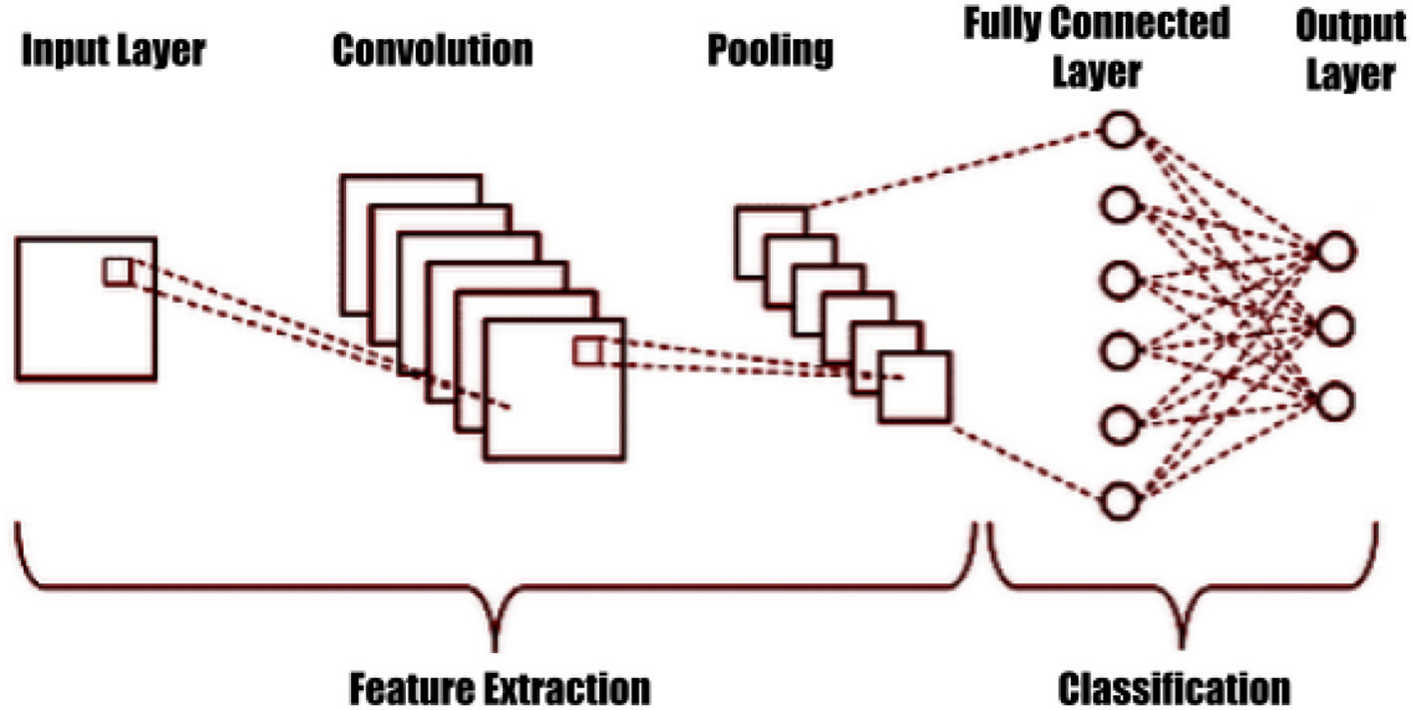
Convolutional neural network structure.

Deep learning (DL) and deep transfer learning are important elements of data science, with applications including statistics and predictive modeling (Iman et al., 2023; Kumar et al., 2023; Mansour et al., 2023b; Sharifani and Amini, 2023). Convolutional neural networks (CNN) are specific architectures for input formats, such as images, and are typically used for image recognition and classification, as shown in Figure 2 (Li et al., 2016; Aloysius and Geetha, 2017; Bharadiya, 2023; Mansour et al., 2023a). These deep neural networks have proven successful in many real-world applications, including image classification, object detection, segmentation, and face detection. Transfer learning takes the classifier layer from a pre-trained CNN and fine-tunes it on the target dataset. This reduces training demands and is a typical technique for using deep CNNs on small datasets. Machine and deep learning-based disease detection is a significant and revolutionary area of medical study. It has become an essential method for the early and precise identification of a variety of illnesses, including but not limited to cardiovascular diseases, cancer, infectious diseases, and neurodegenerative diseases like Alzheimer's. For example, Lei et al. (2021) explored the diagnosis of early Alzheimer's disease using dynamic high-order networks, Wang et al. (2022) addressed the important task of semi-supervised segmenting brain stroke lesions using a consistent perception generative adversarial network (CPGAN), Wang et al. (2019) presented an ensemble approach based on 3D Densely Connected Convolutional Networks (3D-DenseNet) for diagnosing Mild Cognitive Impairment (MCI) and Alzheimer's Disease (AD), and Zuo et al. (2021) proposed a novel multimodal representation learning and adversarial hypergraph fusion (MRL-AHF) framework for Alzheimer's disease diagnosis using complete trimodal images. When analyzing massive datasets, such as clinical data, biomarker data, and medical imagery, machine learning makes use of algorithms and computer capacity to find tiny patterns and predictive signals that would escape human observation. By enabling early intervention, optimizing treatment regimens, and cutting healthcare expenditures, this technology holds enormous promise for bettering patient outcomes. Additionally, as healthcare technology develops, machine learning's role in illness detection is expected to expand, bringing with it fresh ideas for providing people all over the world with healthcare that is more accurate, individualized, and accessible.

This research is the first to use lip mucous images for anemia detection in the literature. The goal of this study is to predict anemia using lip mucous images and to confirm the feasibility of detecting anemia in a non-invasive way. This is accomplished by employing CNN transfer learning. Well-known CNN model, namelys Xception, MobileNetV2, VGG16, and ResNet50, are used for classification of the dataset that contains two lip image classes: anemic and healthy. The classification models were evaluated using metrics: accuracy, recession, recall, and F1 score. The work is of importance because it makes use of AI through DL, which is frequently employed for medical diagnosis and prediction (Kononenko, 2001; Jiang et al., 2017; Christodoulou et al., 2019; Alballa and Al-Turaiki, 2021). This study also provides a non-invasive technique that is more practical and available to patients than blood testing, particularly in places where these tests are not easily accessible. Early diagnosis of anemia is essential since it can stop major health effects including fatigue, weakness, and a weakened immune system. Additionally, creating and training ML algorithms to interpret photos of lip mucous is a low-cost screening technique in regions with a shortage of healthcare resources. Additionally, the possibility of bias or human mistake in the diagnosis of anemia is decreased by the objective examination of lip mucous pictures by DL algorithms. Finally, if confirmed, the DL algorithm may be used to rapidly and reliably assess a huge volume of lip mucous pictures, thus improving the effectiveness of anemia screening programs. The strategy described in our work shows potential for significantly increasing the accuracy and sensitivity of anemia diagnosis when compared to prior studies and standard diagnostic methods. This growth may be attributed to several factors, including the application of DL algorithms with features selection, the inclusion of various data types and demographic data, a huge and diverse dataset, and careful evaluation using performance measurements. Additionally, the non-invasiveness, cost, accessibility, and capabilities of lip mucosa analysis should lead to better patient compliance and quicker anemia detection. Even though these are crucial steps in demonstrating its superiority in actual clinical practice, thorough validation, comparisons with other diagnostic procedures, and clinical trials are insufficient on their own to substantiate these claims.

This article is organized as follows: Section 2 is a discussion of non-invasive approaches for detecting anemia and CNN approaches for anemia detection as well. Section 3 explains the methodology: system design, image processing, and CNN models. The results are given in Section 4. Discussion is presented in Section 5. A conclusion is presented in the final Section, as well as avenues for further research.

## 2. Related work

This section reviews previously used non-invasive anemia detection methods based on conjunctiva and fingertip analyses, and DL models.

Magdalena et al. (2022) used DL techniques, specifically CNN, to carry out conjunctival image-based anemia detection. With the use of the palpebral conjunctiva image and the CNN approach, it was possible to discern between normal and anemic situations with better precision. In total 2,000 photographs of the palpebral conjunctiva, which included anemia and normal circumstances, were used in the investigation. The dataset was then separated into 400 photos for model testing, 160 images for validation, and 1,440 images for training. The study's best accuracy was 94 percent, with average values for precision, recall, and F score of 0.94, 0.94, and 0.93 correspondingly.

Zhao et al. (2022) developed and validated a DL algorithm to predict Hgb values and screen for anemia using ultra-wide-field (UWF) fundus conjunctiva image images. Their study was carried out at the Peking Union Medical College Hospital (Zhao et al., 2022). The dataset was constructed using Optos color photographs that were inspected between January 2017 and June 2021. It was created to use UWF images and ASModel UWF. Its performance was assessed using mean absolute error (MAE) and area under the receiver operating characteristics curve (AUC). To create a visual representation of the model, saliency maps were created. The prediction task's MAE was 0.83 g/dl (95 percent confidence interval: 0.81–0.85 g/dl), and the screening task's AUC was 0.93 for ASModel UWF (95 percent CI: 0.92–0.95). The model tended to concentrate on parts of the retina that were missed by non-UWF imaging, such as the regions around the optic disc and retinal arteries.

Rivero-Palacio et al. (2022) used YOLO v5 to create a smartphone app for anemia screening. They made advantage of a conjunctiva image collection received from the Universidad Peruana Cayetano Heredia, which features images of young children and their blood test prognosis. Although YOLO v5 performs well when used on a computer, its performance is diminished when used in a mobile application. Despite this, the app's ability to identify anemia has a sensitivity of 0.71 and a specificity of 0.89.

Huang et al. (2021) developed CycleTrack, which is a new brand DL multi-cell tracking algorithm that can accurately count blood cells from capillaroscopic movies. CycleTrack is designed to mimic the characteristics of capillary blood cell flow and combines the two straightforward online tracking techniques, SORT, and CenterTrack. Displacement vectors in two opposing temporal directions between subsequent frames are used to follow blood cells. With this method, blood cells are accurately tracked even when they move quickly and change shape. On test videos, the suggested model surpasses alternative baseline trackers, scoring 66.3 percent MOTA and 75.1 percent ID F1. With minimal effort investment, CycleTrack achieves an average cell counting inaccuracy of 3.42 percent among eight 1000-frame test movies, compared to original CenterTrack and SORT's 6.55 percent and 22.98 percent.

Kasiviswanathan et al. (2020) developed a noninvasive approach for determining an individual's anemic state by assessing their hemoglobin (Hb) level. This model was developed using data gathered from 135 participants, of which 108 were classed as part of the training group and the remaining 27 as part of the test group. The data from the test group was used to test the model, which is based on the Ridge Regression algorithm. Using a computerized image of the lower palpebral conjunctiva and basic information like age, sex, height, weight, and BMI, it is possible to predict a person's hemoglobin level. The Pearson correlation coefficients between the measured hemoglobin value and the anticipated hemoglobin value are 0.722 and 0.705 for training and testing, respectively. The predicted values were closer to the values measured by the standard invasive methods.

Mitra et al. (2021) proposed a unique non-invasive algorithm that identifies anemia using human nails. They employ computer vision, ML, and DL concepts in their methodology, and only on the basis of those data they predicted the degree of anemia for the participant. They suggest a method that is fully real-time, and this system was able to produce results.

Golap et al. (2021) created a model for the estimate of a non-invasive hemoglobin and glucose level using photoplethysmogram (PPG) characteristics retrieved from fingertip videos recorded on a smartphone based on multigene genetic programming (MGGP). The PPG signal was created by processing the videos. A total of 46 features have been recovered by analyzing the PPG signal, its first and second derivative, and using Fourier analysis. The best characteristics were then chosen using a genetic algorithm and a correlation-based feature selection approach. Finally, a symbolic regression model based on the MGGP was created to estimate glucose and hemoglobin levels. Several traditional regression models were also created utilizing the identical input condition as the MGGP model in order to compare the performance of the MGGP model. By calculating various error measurement indices, a comparison between MGGP-based models and conventional regression models was conducted. Selected characteristics and symbolic regression based on MGGP were used to find the best results (0.304 for hemoglobin and 0.324 for glucose) among these regression models.

Haque et al. (2021) suggested a unique non-invasive method based on PPG signal to assess blood hemoglobin, glucose, and creatinine levels (DNN). Using a smartphone, 93 individuals' fingertip videos were gathered. From each movie, the PPG signal is created, and 46 distinguishing features are then taken from the PPG signal, its first and second derivatives, and Fourier analysis. Age and gender are also taken into consideration because of their significant impacts on hemoglobin, glucose, and creatinine. To reduce redundancy and over-fitting, the best features have been chosen using genetic algorithms (GA) and correlation-based feature selection (CFS). Finally, using the chosen characteristics, DNN-based models were created to predict the blood levels of hemoglobin, glucose, and creatinine. The method offers R_2_ = 0.922 for Hb, R_2_ = 0.902 for Gl, and R_2_ = 0.969 for Cr as the best-estimated accuracy.

Moral and Bal (2020) utilized CNN to calculate total hemoglobin concentration. The developed frequency-domain multidistance approach (FDMD), based on a non-contact oximeter, provided data on total hemoglobin. They used a non-contact, frequency-domain multi-distance FDMD-technique-based oximeter to record the signals from 24 healthy volunteers aged 20 to 42 with gold standard hemoglobin (Hb) levels of 9 to 16 g/dL. Using the scalogram, which shows the absolute value of a signal's continuous wavelet transform (CWT) as a function of time and frequency, one-dimensional 155 signals were transformed into images. Their research suggests using transfer learning to estimate total hemoglobin concentration using enhanced data from a deep CNN model developed beforehand called AlexNet. For both 20 and 50 MHz, the experimental findings used transfer learning and AlexNet to reach up to 87.50% test accuracy.

Most of previous methods detected anemia using data such as conjunctival images, human nails, and fingertips. ML, DL, and CNN were used in some of them to develop invasive and noninvasive methods for detecting anemia. The performance still needs to be improved through applying and using new data and simple methods. This study is important as it is the first to deal with lip mucous images for predicting anemia using CNN. The study's use of DL, which is frequently employed in the medical field for prediction and diagnosis, is what provides it its significance (Kononenko, 2001; Jiang et al., 2017; Christodoulou et al., 2019; Alballa and Al-Turaiki, 2021).

## 3. Methodology

**Figure 3:**
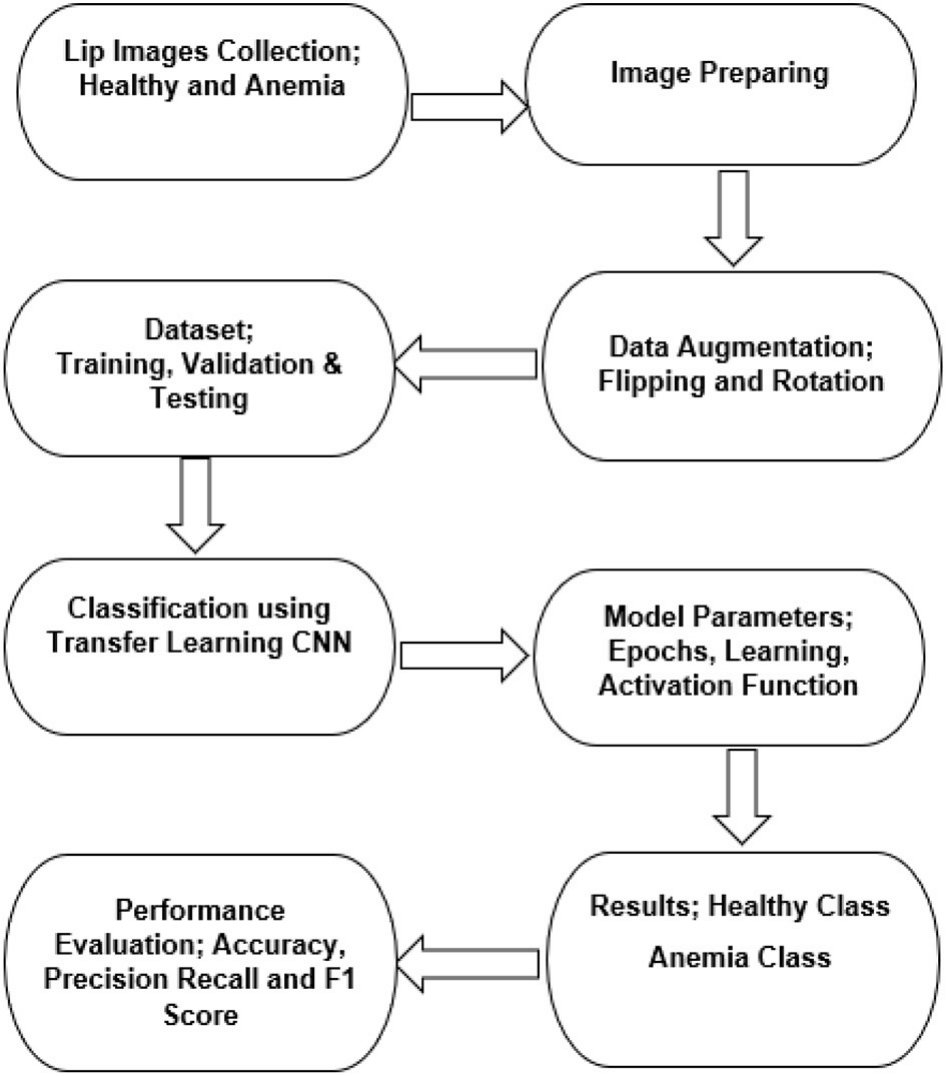
Study flow chart.

The classification problem is to detect anemia using a collected lip image dataset. The study starts by building the dataset which has two lip images types: healthy and anemic. Following this, digital images processing was performed and CNN transfer learning models for classification were then used and evaluated to establish the best model. Figure 3 shows the flow chart of the study.

### 3.1. Data collection

Participants were recruited between November and December 2021, following Sakarya University's Ethical Approval (E-71522473-05.01.04-74571-458). Participants from the Pamukova District of Sakarya Province made up the study group. A total of 138 people took part in the study, with 100 women and 38 men. Anemia was diagnosed in 23 women and six men according to WHO standard values (Organization, 2011). The convenience sampling approach was used to determine the study group (Patton, 2005). Table 1 shows the demographic characteristics (gender and age ranges).

**Table 1:** Participants demographic values.

**Variable**		**Frequency**	**Percentage**
Gender	Female	100	72.46
	Male	38	27.54
	18–30	53	38.4
Age	30-50	36	26
	50	49	35.6

**Figure 4:**
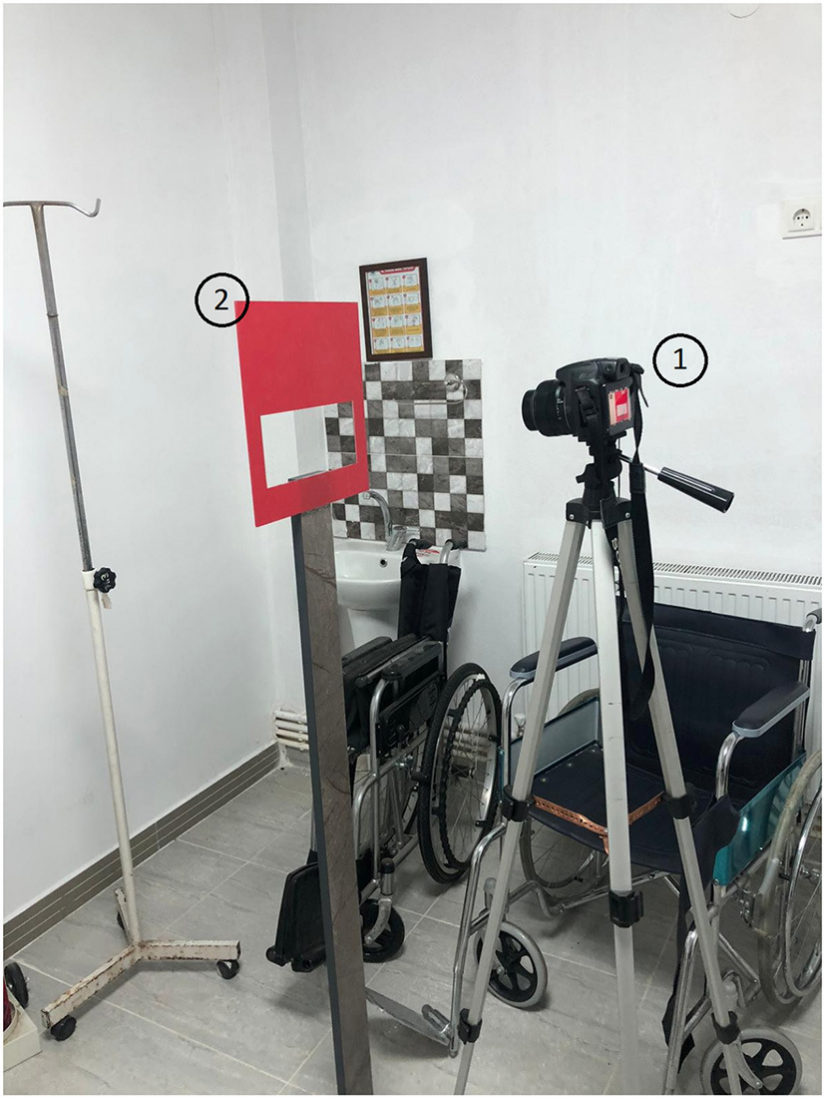
System setup: (1) Camera, (2) Frame.

The experimental setup shown in Figure 4 was designed to measure the facial features of the participants, and it is designed to display only the lip area with the help of an adjustable frame. A camera captured a high-resolution image (4896 × 2752) of each participants. Data collection was carried out by a team of experienced medical doctors.

### 3.2. Image processing and data augmentation

**Figure 5:**
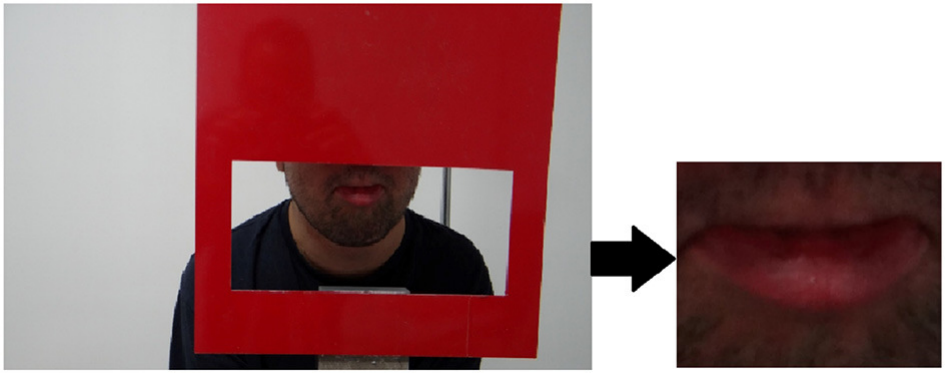
Example raw image and segmented image in RGB space.

**Figure 6:**
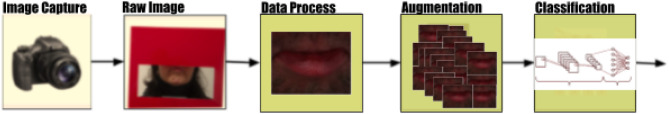
Flowchart of images processing & classification.

To process and analyze data, a custom Python application was created. Firstly, the participant's lip contour was determined using corner detection, threshold value, and framing. The framed digital image was then converted to rgb formats as shown in Figure 5, after which it was analyzed and classified using CNN structures (see Figure 6).

DL requires large amounts of data, however, data is not always available in all cases (Shorten and Khoshgoftaar, 2019). Therefore, the data extension was applied to improve data diversity by making minor modifications to existing data copies or by creating synthetic data from existing data. Some of the techniques used for data expansion are rotation, flipping, shear, brightness or contrast changing, cropping, scaling, and saturation. In this research, horizontal and vertical flipping and rotation were applied to dataset. A total of 1,380 images were developed using data augmentation techniques.

### 3.3. Transfer learning and CNN models

In recent years, transfer learning, a method which learns from similar tasks, has drawn increasing attention. Transfer learning uses a pre-trained CNN that is optimized for a single task. Four common CNN architectures were used in this research with pretrained weights. VGG16, ResNet50, MobileNet, and Xception are used to categorize lip mucous images as healthy rather than anemic. The initial model incorporates data from pre-trained CNN models to detect anemia from lip images. For CNN models, the input image size is set at 224 × 224. Images were pre-processed before being sent via the CNN convolutional layer. The CNN architectures were used directly from keras and tensorflow libraries with their trained weights. The layers and filter sizes were used without any changes. Global average pooling 2D layer was added to convert the features to a single element vector per image and the sigmoid activation layer function was used in the final layer.

For CNN algorithms to be practical and successful in addressing real-world issues, they must perform well (Hossin and Sulaiman, 2015; Flach, 2019). High-performing algorithms may produce more precise predictions, analyze data more quickly, scale to handle big datasets, and be easier to understand, which improves outcomes and decision-making. In ML, classification models are frequently used to forecast outcomes based on a set of traits. There are a number of widely used measures available to assess the performance of such models. The problem's nature, class balance, and the model's intended result all influence the measure that is used (Prati et al., 2011; Santafe et al., 2015; Tharwat, 2021). A straightforward statistic called accuracy counts how many of the model's predictions were accurate (Folorunso et al., 2022). When there is a class imbalance in the data, it might not be the best option. When minimizing false positives, precision indicates the percentage of true positives across all positive predictions provided by the model (Juba and Le, 2019; Miao and Zhu, 2022). Contrarily, recall assesses the proportion of real positives among all of the actual positive instances in the data and is helpful when the objective is to reduce false negatives (Ali et al., 2022; Miao and Zhu, 2022). F1 score is the harmonic mean of precision and recall and is useful to balance the importance of both (Hossin and Sulaiman, 2015). Finally, by showing the quantity of true positives, true negatives, false positives, and false negatives for the specified model, the confusion matrix offers a more thorough understanding of the performance of the model than any single statistic (Haghighi et al., 2018; Liang, 2022). A DL model's performance evaluation is crucial to determining its efficacy and pinpointing areas for development. The model should be evaluated using a variety of metrics, and while selecting a statistic, the problem's context should be taken into account.

## 4. Results

The CNN architecture was applied to detect anemia in lip mucous images. Experiments were performed in the Python programming language, as well as its libraries Keras, sci-kit, and Tensorflow. Before the classification process, the images were prepared and augmented in order to improve the accuracy of the model and reduce the degree of model overfitting.

The numbers of epochs, hidden layers, hidden nodes, activation functions, dropouts, learning rates, and batch size are used to fine-tune the model. Hyper parameter tweaking has an impact on the performance of the model. Hyper parameters used are shown in Table 2.

**Table 2:** Parameter setting.

**Parameter**	**Value**
Image size	224*224
Convolutional and max pooling	CNN transfer learning
Learning rate	0.00001, 0.0001
Epochs	100
Activation function	Relu, Sigmoid
Dropout rate	0.20, 0.25

The test-split python function was used to split the dataset. In total 70% of the images was randomly allocated as training and 30% as test data. Four CNN algorithms were pre-trained. For all CNN's models, the input layer uses 224 by 224 images, 100 epochs, 16 batch size, 0.0002 learning rate, early stopping mode, and adam optimizer. The models carry out a max-pooling operation in each pooling layer with a modified pool size and the network's RELU function. A sigmoid activation function was used for the two output classes. Hyper parameters including learning rate and epoch size were changed during the network's training phase. To maximize the targeted performance measurement, the learning rate was tested at various settings. The total number of images in the collection was used as the basis for the validation procedure. The accuracy improved as different epochs and batch sizes were adjusted.

**Figure 7:**
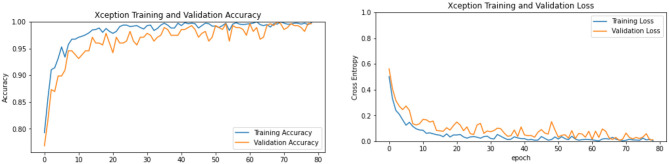
Training and validation, accuracy and loss of Xception.

The effectiveness of a model was assessed and validated using training, testing, and validation techniques. The training and validation accuracy as well as the loss of anemia detection for the best model's results are shown in Figure 7. As seen in Table 3, the confusion matrices shows the predicted classes and their disturbance.

**Table 3:** Confusion matrices.

**Xception**		**VGG16**		**MobileNetV2**		**ResNet50**	
TP = 217	FP = 1	TP = 210	FP = 8	TP = 215	FP = 7	TP = 215	FP = 8
FN = 1	TN = 57	FN = 8	TN = 50	FN = 3	TN = 51	FN = 3	TN = 50

Table 3 shows the confusion matrix after it has been calculated. All classes' model performance is precisely measured. The accuracy is described in the following manner.


(1) $$\begin{array}{l} Accuracy=\frac{TP+TN}{TP+TN+FP+TN} \end{array}$$

**Figure 8:**
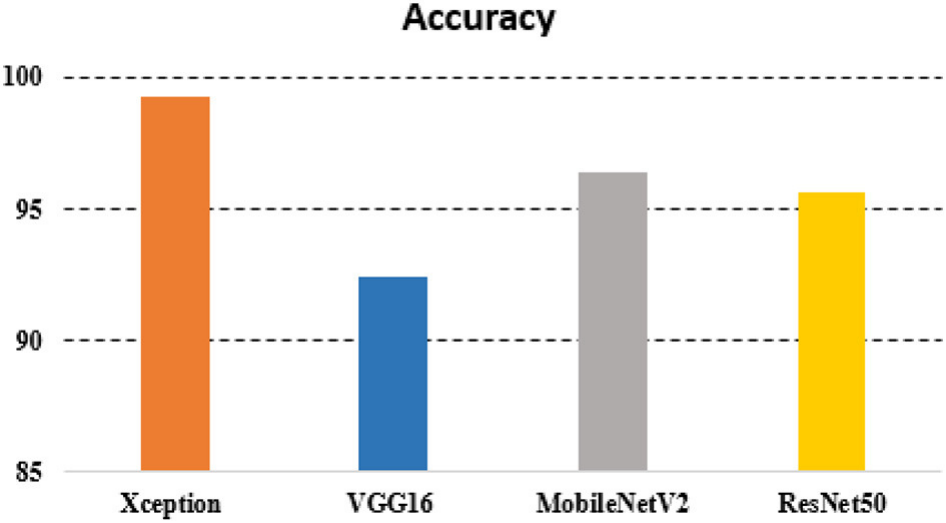
Accuracy chart.

**Figure 9:**
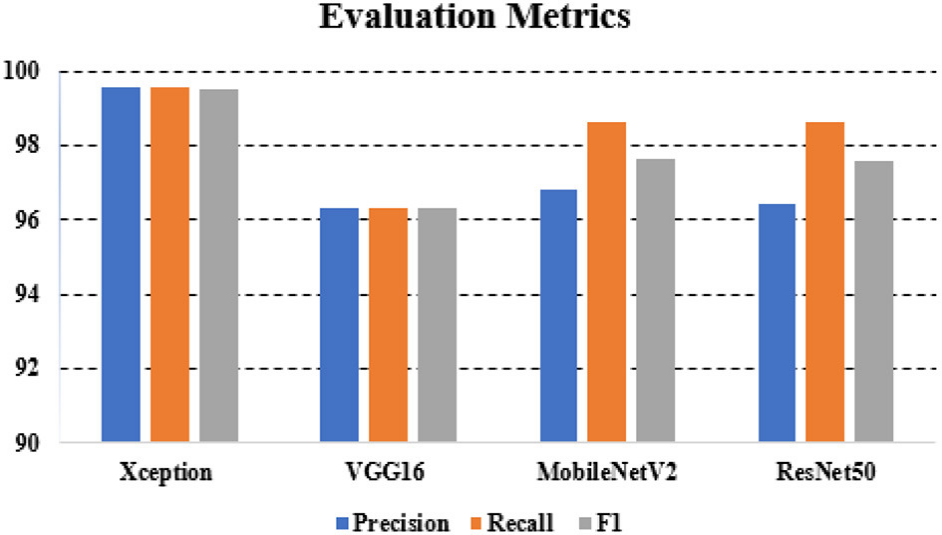
Evaluation metrics for CNN architectures.

The overall accuracy was 92.39% with VGG16, 96.38% with MobileNet, 95.65% with ResNet50, and 99.28% with xcpetion. Table 4 and Figure 8 presents the accuracies of the CNN architectures. The Xception algorithm was shown to be more accurate than the other models. Table 5 and Figure 9 shows the CNN Models assessment parameters. In this study, these primary criteria were precision, recall, and F1 score for the two predicted classes.

**Table 4:** Accuracy table.

**Algorithm**	**Accuracy(%)**
VGG16	92.39
ResNet50	95.65
MobileNetV2	96.38
Xception	99.28

**Table 5:** CNN models assessments.

	**Precision**	**Recall**	**F1 score**
Xception	99.54	99.54	99.5
VGG16	96.33	96.33	96.32
MobileNetV2	96.84	98.62	97.63
ResNet50	96.41	98.62	97.6

## 5. Discussion

In this study, a CNN model was used to detect anemia using lip mucous images. The findings of this study are consistent with other studies that used DL models for invasive and non-invasive methods for predicting anemia. The performance of CNN models in predicting anemia was evaluated using accuracy, specificity, precision, recall, and F-measure. The accuracy performances of the studied models were high and significant. Xception reported the best accuracy, sensitivity, and F1 measures for anemia prediction.

**Figure 10:**
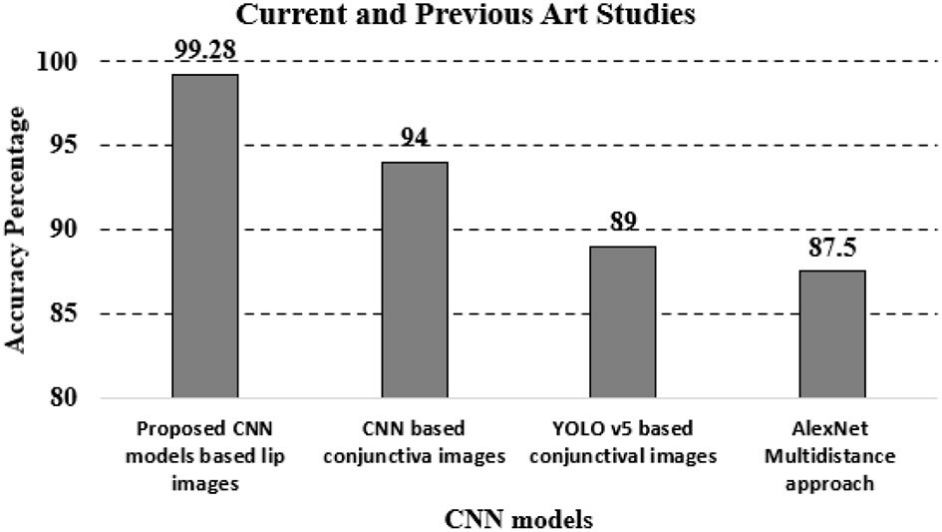
Current and previous studies.

Limited methods were found in the literature to detect anemia. When comparing the test results obtained from this application with laboratory data, the K-means clustering approach, for instance, revealed an accuracy of 90% when utilized to carry out conjunctival pallor image-based anemia identification (Sevani et al., 2018). In order to estimate HGB levels non-invasively, ANN was used to evaluate photos of fingertips obtained with a smartphone camera. The model's hemoglobin levels and the gold standard hemoglobin levels were found to be correlated with 0.93 (Hasan et al., 2018). CNN techniques were used to carry out conjunctival image-based anemia detection with an accuracy of 94% (Magdalena et al., 2022). YOLO v5 was used to detect anemia using conjunctiva image collection with a sensitivity of 0.71 and a specificity of 0.89 (Rivero-Palacio et al., 2022). AlexNet was used to calculate total hemoglobin concentration by developing a frequency-domain multidistance approach, based on a non-contact oximeter, and provided data on total hemoglobin with accuracy of 87.50% (Moral and Bal, 2020). Compared to these methods that used CNN and DL in general for anemia detection, the current study is a noninvasive method that uses CNN models to detect anemia with higher accuracy: 99.28% using xception (see Figure 10). This new method is simple and can be developed for real-time anemia detection.

Lip pallor, which is characterized by the pallor or loss of natural color in the lips, has substantial advantages as a non-invasive diagnostic site when compared to other human body parts. First off, because the lips are so visible and accessible, there is no need for specialized equipment or invasive treatments to inspect them. Lip pallor is a practical alternative for diagnostic methods because of its accessibility, allowing rapid and little intrusive patient evaluations. On the lips, there is also a dense vascular network with many tiny blood vessels near to the surface. Variations in blood flow and oxygenation, which typically manifest as changes in lip color, may be immediately seen because of the vascular richness. Because of the close association between lip color and the circulatory system, the lips are ideal for diagnosing blood-related illnesses including anemia. They provide current health-related information about a patient.

Because lip pallor analysis is non-invasive, patient comfort and compliance are improved. Whether it comprises basic eye exams or more advanced imaging methods, the procedure is safe and suitable for people of all ages. Due to the fact that it is typically socially acceptable in all cultures to examine one's lips, this technique also complies with moral principles and cultural norms. A patient may be more eager to take part in lip-based diagnostic procedures if they feel accepted. Because of developments in imaging technology, it is now feasible to do exact color and texture analyses of the lips in high-resolution pictures. This level of specificity ensures that lip pallor analysis will always be a useful and economical screening approach in medical settings, making it an important tool in the field of non-invasive diagnostics. It is necessary for spotting minute indications of disorders like anemia.

Given that DL is still in its infancy when being used in medical research, this finding also makes an important addition to that field. This study may serve as a starting point for future work on creating ML-based tools for anemia detection and diagnosis, which might result in even more precise and potent diagnostic equipment. Our findings suggest that deploying CNN techniques for anemia detection will help in diagnosing using classification, which will in turn aid in the development of effective preventive interventions. Thus, this research not only addresses the integration of innovative technology for the prediction and diagnosis of low hemoglobin levels but also supports the medical system through assessing the prediction power of several CNN algorithms.

## 6. Conclusions

Images of lips were gathered and compiled as a dataset for this paper. The CNN models are subjected to the methods of data augmentation, dataset preprocessing, training, and testing. In comparison to other techniques, CNN's evaluation metric parameters are greater and more extensive. With a 99.28% accuracy, the proposed research is suitable for deployment.

This work has established a link between anemia detection and lip mucous images. The conjunctiva is used in general practice. A method that is easy to use and can be classified on the lip mucous was demonstrated for the first time. In the future, augmented data will be used for online classification and the detection of anemia using a mobile application.

## Data availability statement

The raw data supporting the conclusions of this article will be made available by the authors, without undue reservation.

## Ethics statement

The studies involving humans were approved by Sakarya University's Ethical Approval (E-71522473-05.01.04-74571-458). The studies were conducted in accordance with the local legislation and institutional requirements. The participants provided their written informed consent to participate in this study.

## Author contributions

SM: Funding acquisition, Methodology, Resources, Writing—original draft. MM: Software, Writing—original draft, Writing—review & editing. TD: Data curation, Supervision, Writing—original draft. MK: Investigation, Methodology, Writing—original draft. CF: Writing—original draft, Methodology, and Supervision.
