# Corrigendum: Non-invasive detection of anemia using lip mucosa images transfer learning convolutional neural networks

**DOI:** 10.3389/fdata.2023.1338363

**Published:** 2023-12-20

**Authors:** Shekhar Mahmud, Mohammed Mansour, Turker Berk Donmez, Mustafa Kutlu, Chris Freeman

**Affiliations:** ^1^Department of Systems Engineering, Military Technological College, Muscat, Oman; ^2^Mechatronics Engineering Department, Sakarya University of Applied Sciences, Serdivan, Sakarya, Türkiye; ^3^Biomedical Engineering Department, Sakarya University of Applied Sciences, Serdivan, Sakarya, Türkiye; ^4^Electronics and Computer Science, University of Southampton, Southampton, United Kingdom

**Keywords:** anemia, image processing, deep learning, classification, convolutional neural network (CNN)

In the published article, there was an error in the author list, regarding the order of author [Shekhar Mahmud]. There was also an author missed off the original author list [Chris Freeman].

The new requested author order is:

Shekhar Mahmud, Mohammed Mansour, Turker Berk Donmez, Mustafa Kutlu and Chris Freeman.

The authors apologize for this error and state that this does not change the scientific conclusions of the article in any way. The original article has been updated.

## Author contributions

SM: Funding acquisition, Methodology, Resources, Writing—original draft. MM: Software, Writing—original draft, Writing—review & editing. TD: Data curation, Supervision, Writing—original draft. MK: Investigation, Methodology, Writing—original draft. CF: Writing—original draft, Methodology, and Supervision.

